# Data of collaborative consumption in online food delivery services

**DOI:** 10.1016/j.dib.2019.104007

**Published:** 2019-05-23

**Authors:** Miguel A. Segura, Juan C. Correa

**Affiliations:** Faculty of Psychology, Fundación Universitaria Konrad Lorenz, Bogotá, Colombia

**Keywords:** Collaborative consumption, Web scraping, Online food ordering, Traffic conditions, Google maps

## Abstract

This data article contains data regarding the collaborative consumption that takes place in an Online Food Delivery Platform that connects restaurants owners, and customers who wish to order meals and receive them at home or office. These data are associated with the article “Evaluation of Collaborative consumption of food delivery services through web mining techniques” [1]. These data are stored in a comma separated value format; that can be easily downloaded from a Mendeley data repository (https://data.mendeley.com/datasets/m9z9hw4nsc/1).

**Table:** Specifications Table

Subject area	*Psychology, business Management and Accounting*
More specific subject area	*Consumer Psychology, Marketing*
Type of data	*Table (Data frame)*
How data was acquired	*We developed an ad-hoc web scraper procedure with the software “Agenty” for retrieving key performance indicators of 787 fast-food providers available at a Colombian website (*https://domicilios.com/bogota*). We used Google Maps API for retrieving expected travel times between the provider's physical location and the customer's addresses. We queried the geographic location of these providers from Facebook, and we set its latitude and longitude coordinates with the “Batch Geocode Tool”. Given the fact that customers' information remains private according to the website information policy, we generated a random sample of geographic points from Google Maps as a valid replacement of real customers' addresses.*
Data format	*Raw*
Experimental factors	*The “Typical Traffic” around each restaurant is characterized following Google Maps visualization (“green” for free traffic flow; “red” for congested traffic flow or “orange” for intermediate traffic flow) for three different moments of rush hours (“mornings”, “noons”, and “afternoons”). The “Name of Provider” refers to the commercial name of each restaurant.*
Experimental features	*Our first experimental factor is the traffic moment or just “Moment”. Moment is a categorical variable with three possible values: “Morning”, “Noon”, and “Afternoon”. “Moment” helps us to identify the typical traffic, as captured by Google Maps API, around each restaurant during rush hours on Saturdays. Preliminary analyses using Google Maps API revealed us those rush hours took place in mornings (between 8 and 10 a.m.), noons (between 12 and 2 p.m.), and afternoons (between 6 and 8 p.m.) in Bogotá City.* *Our second experimental factor is the typical traffic of rush hours in the following three categories: free or green traffic (G), average or orange traffic (O), and heavy or red traffic (R). We generated letter triads that allowed us to characterize the typical daily traffic. Thus, for example, the sequence “R-O-G” means that the typical traffic changes from “red” in the morning to “orange” at noon and “green” in the afternoon, describing a place where traffic conditions improve as time passes.*
Data source location	*Bogotá, Colombia. Latitude and Longitude coordinates of both restaurants and customers are provided in the table.*
Data accessibility	*The data is available at*https://data.mendeley.com/datasets/m9z9hw4nsc/1
Related research article	*J.C. Correa, W. Garzón, P. Brooker, G. Sakarkar, S.A. Carranza, L. Yunado, A. Rincón, Evaluation of collaborative consumption of food delivery services through web mining techniques, J. Retail. Consum. Serv. 46, 2019, 45–50*[1].

**Table:** 

**Value of the Data** • The data present a novel set of records that allows the evaluation of the relationship between performance indicators of Online Food Delivery Services and traffic conditions as captured by Google Maps API. • The data was collected by means of computational methods such as “APIs” (application programming interface) and “web scraping” that allow the automatic collection of available public information from a Colombian website of Food Delivery, and can be easily generalizable to worldwide websites such as “just-eat”, “UberEATS”, “FoodPanda”, etc. • The records provide another perspective to retrieve relevant data of collaborative consumption in online food delivery services without using questionnaire-based methods that are common in consumer behavior research [2].

## 1 Data

The data set was built with the purpose of evaluating the impact of traffic conditions on key performance indicators of a sample of 787 restaurants with food delivery services in Bogotá City. The data set includes the physical location of both restaurants and 4296 customers in Bogotá city, as well as the key performance indicators of restaurants and their traffic descriptions, as captured by Google Maps API during three moments with traffic rush hours of Saturdays.

## 2 Experimental design, materials, and methods

We developed and used an advanced web scraper named “Agenty” (https://www.agenty.com/) to extract five key performance indicators of 787 restaurants with food delivery services in Bogotá City through a local website (https://domicilios.com/bogota).

The first performance indicator, “cost of the delivery”, reflects the amount of money in Colombian pesos charged for dispatching the food from the restaurant to the customer. The second indicator was the expected delivery time, which is the providers' declared times (in minutes and seconds) to deliver their orders to their customers. The third indicator, “minimum charge ordering”, is the minimum charge in Colombian pesos required for providers to deliver their orders to the customer. The fourth indicator is the “number of comments” that each restaurant received from customers by the date we retrieved the information. The number of comments is the most relevant indicator of transactions volume. This number, in no way, equals the total number of customers who ordered a service. However, it shows the number of customers who ordered some service and left a positive or negative comment about it. As such, the number of comments is necessarily lower than the total of customers who made a transaction with the food provider, but it is sensibly informative about the customers who care about allowing other customers know their experience with the food provider, which shows a conceptual match with the idea of collaborative consumption. We calculated a fifth indicator, called “delivery time fulfillment” (DTF). DTF is the difference between providers own declared delivery times (publicly available on the website) and the expected travel time provided by Google Maps API [3].

The geographic location of food providers was queried from Facebook as it conveys a behavioral indicator that shows that restaurant owners are interested in interacting with their clients through this social network. As customers' information is private according to the website information policy, we generated a random sample of geographic points from Google Maps as a valid replacement of real customers' addresses.

The analyses of these data can be easily reproduced by downloading and running an R script that is available at https://github.com/jcorrean/WebMining-OFD. Interested readers can find additional theoretical guidance in our published paper [1].
